# Supplementary material for: Sexually dimorphic renal expression of Klotho is directed by a kidney-specific distal enhancer responsive to HNF1b
Source: bioRxiv. 2024 Mar 4:2024.02.29.582831. Preprint. [Version 1] doi: 10.1101/2024.02.29.582831 (PMC10962737; doi:10.1101/2024.02.29.582831)
Supplement: Supplement 1 [file media-1.pdf]

Supplemental data

Sexually dimorphic renal expression of Klotho is directed by a kidney-specific distal enhancer responsive to HNF1b

Jakub Jankowski[1, *], Hye Kyung Lee[1], Chengyu Liu[2], Julia Wilflingseder[3], Lothar Hennighausen[1]

1 Section of Genetics and Physiology, Laboratory of Cellular and Molecular Biology, National Institute of Diabetes and Digestive and Kidney Diseases, US National Institutes of Health, Bethesda, Maryland, 20892, USA

2 Transgenic Core, National Heart, Lung, and Blood Institute, US National Institutes of Health, Bethesda, MD 20892, USA

3 Department of Physiology and Pathophysiology, University of Veterinary Medicine Vienna, Veterinärplatz 1, 1210 Vienna, Austria

Figure and table legends

Supplemental Fig. 1 No eRNAs at are detected at the *Klotho* enhancer locus. ChIP-seq and RNA-seq were performed as described in the materials and methods section. Alignment of the RNA-seq to the PolII tracks indicates absence of enhancer RNA.

Supplemental Fig. 2 Mouse weight is not impacted by enhancer 1 knockout. Random litters of male, female, WT and E1 KO mice (n=8) at 3-4 months old were weighed. Unlike other Klotho knockout models, there is no significant difference in weight and mice survive into adultohood. Bar = SEM.

Supplemental Fig. 3 There is no indication of female sex hormones impacting Klotho levels. Renal Klotho mRNA was measured in 3-week old mice (A, n=4, data excluded due to undetectable *Klotho* expression) and in 8-week old mice ovariectomized at 3-weeks old (B, n=4). Sexual dimorphism of Klotho is detectable in prepubescent mice and ovariectomy does not impact mRNA levels. E2 deletion in females has no effect on Klotho expression (C, n=4.) Bar = SEM

Supplemental Fig. 4 E1 deletion does not impact blood biochemistry. Serum biochemistry panel was performed as described in materials and methods section. No differences were observed between any of the experimental groups. A-I – n=4. Bar = SEM

Supplemental Fig. 5 Control loci for RNA-seq analysis; housekeeping genes expressed in the kidney, not directly related to Klotho and displaying similar expression levels across genotypes.

Supplemental Fig. 6 Representative photographs of injured kidneys and contralateral controls 28 days after unilateral kidney ischemia reperfusion surgery in male and female WT and E1/E2 mice. Bar = 50µm.

Supplemental Table 1 sgRNA and primer sequences used to construct and genotype E1, E2 and E1/E2 mice.

Supplemental Table 2 Klotho E1 and E2 deletion sequences.

Supplemental Spreadsheet 1 Gene counts and differentially expressed genes in WT and E1 KO male and female mice.

Supplemental Figure 1

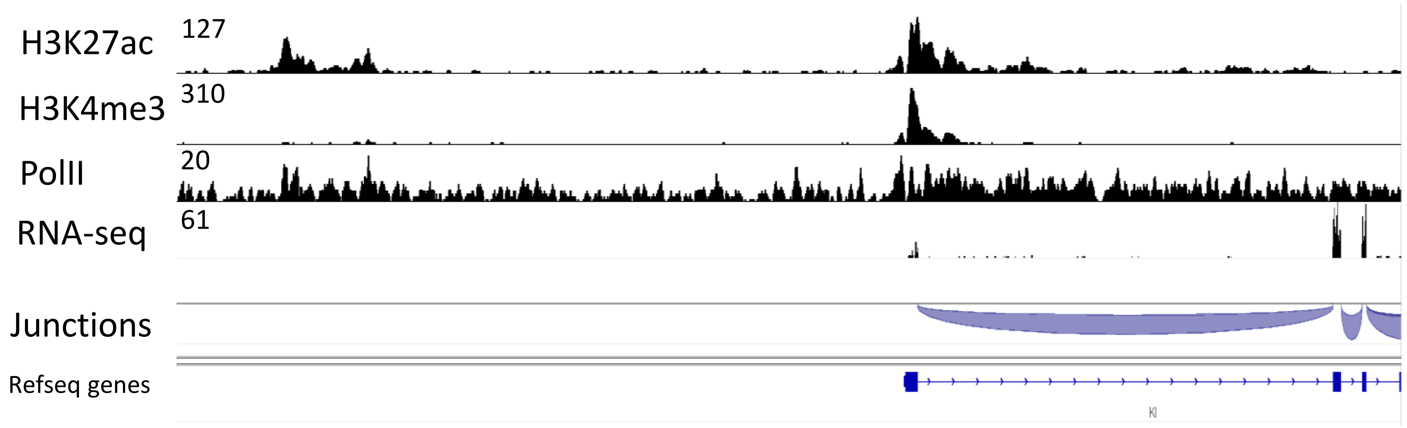

Supplemental Figure 2

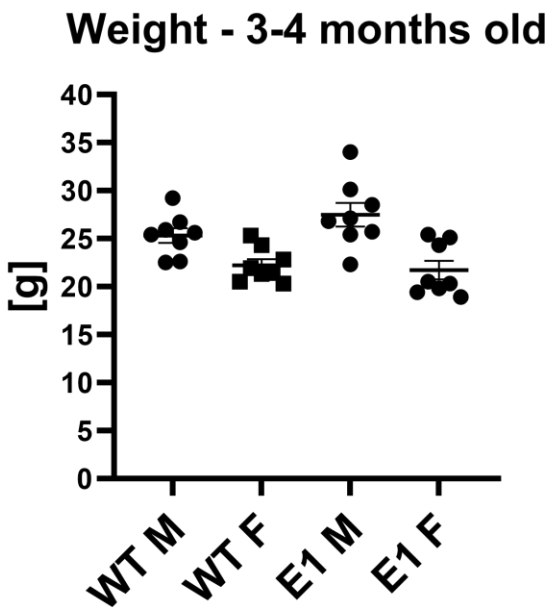

Supplemental Figure 3

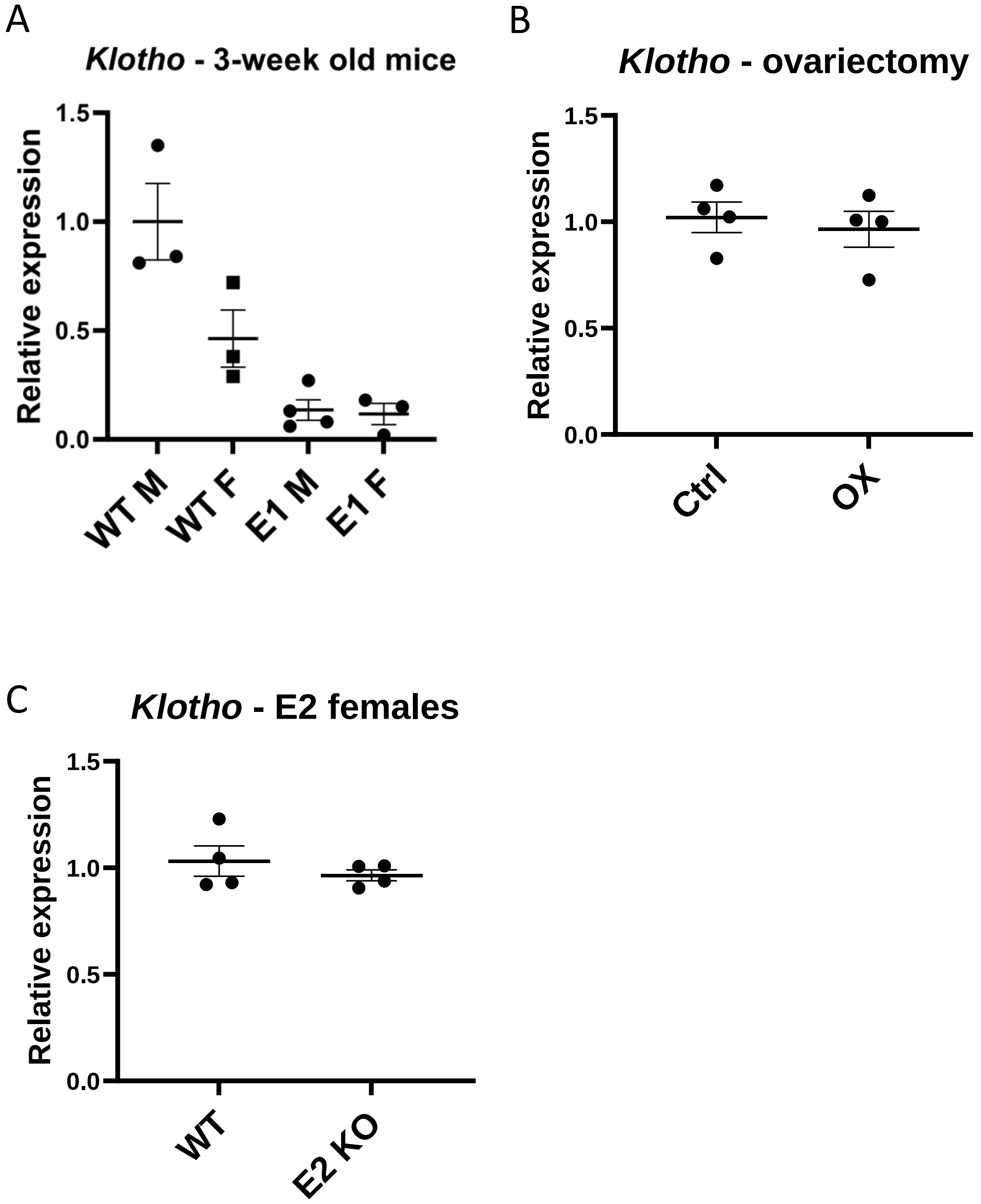

Supplemental Figure 4

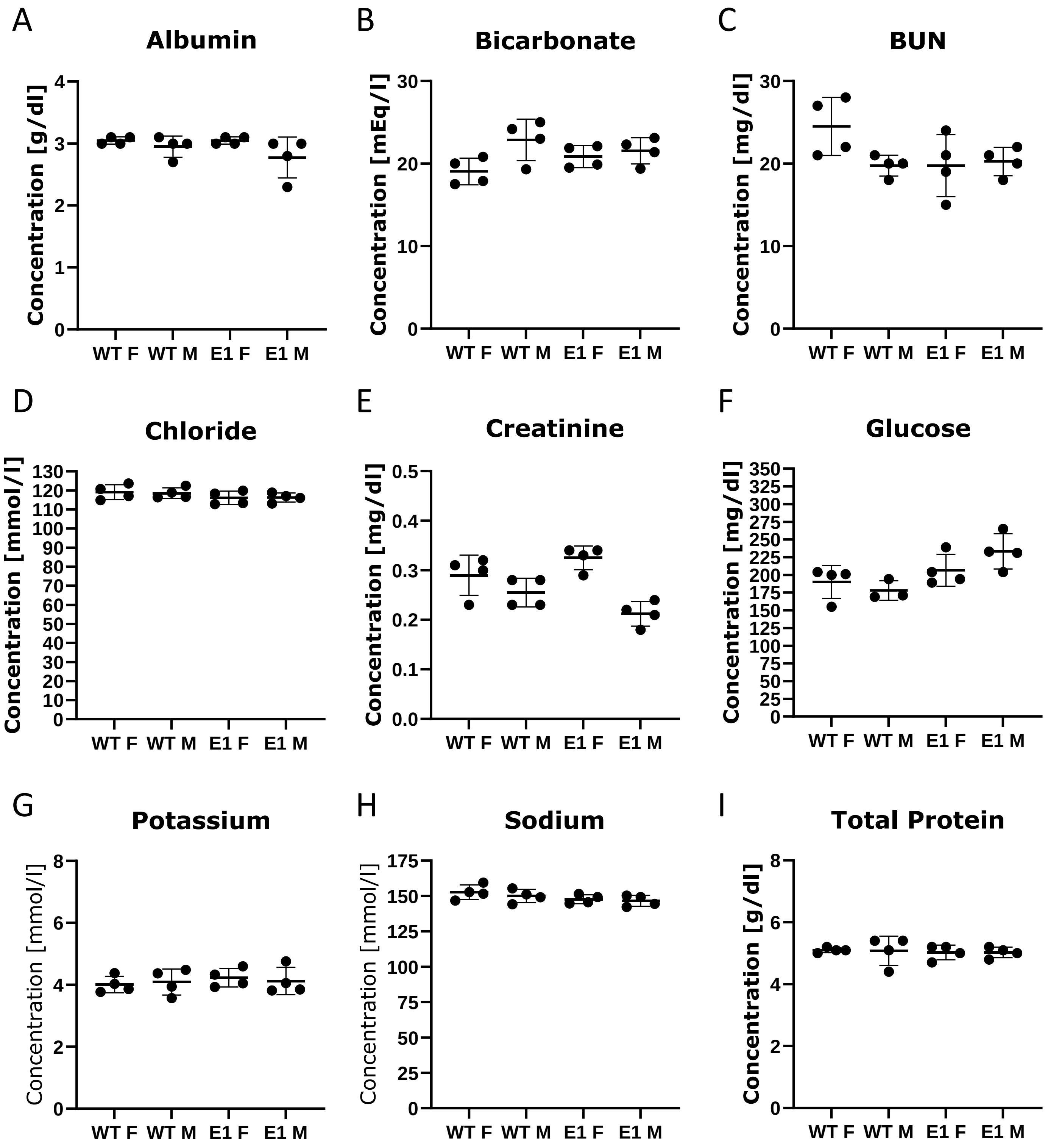

Supplemental Figure 5

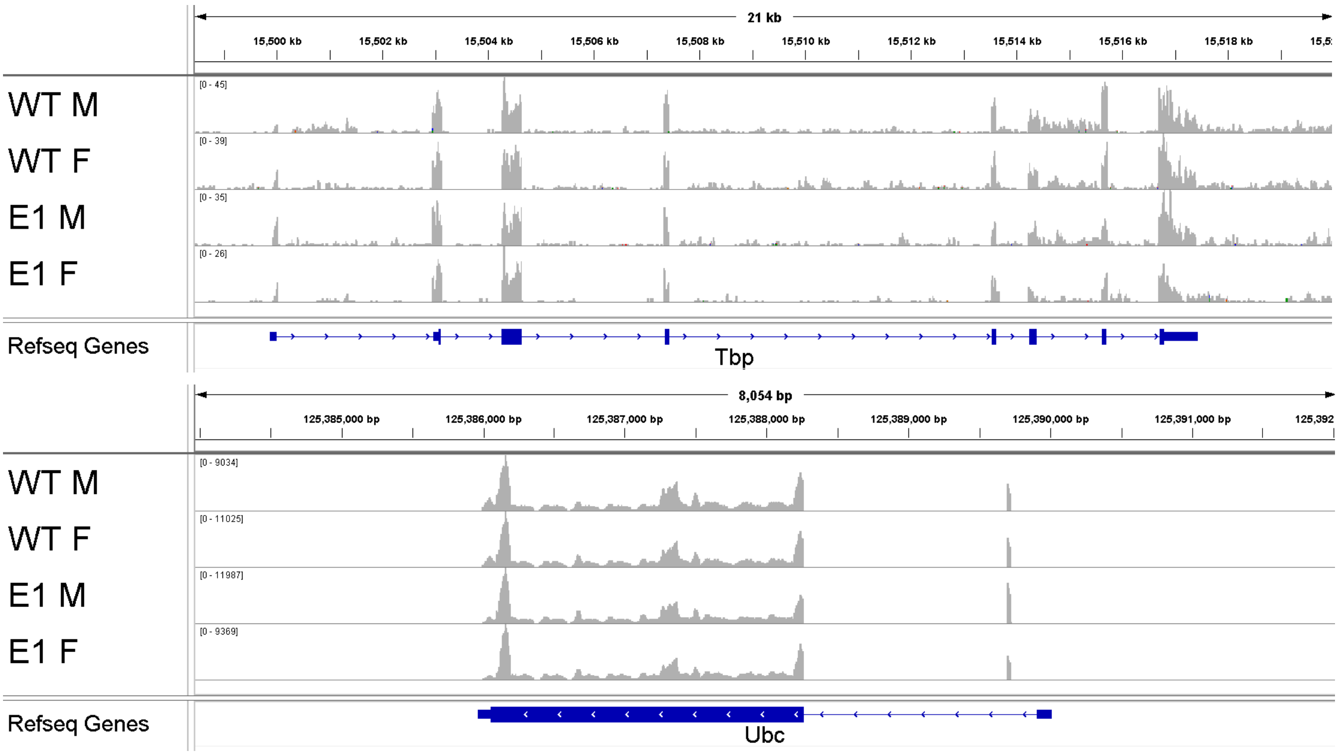

Supplemental Figure 6

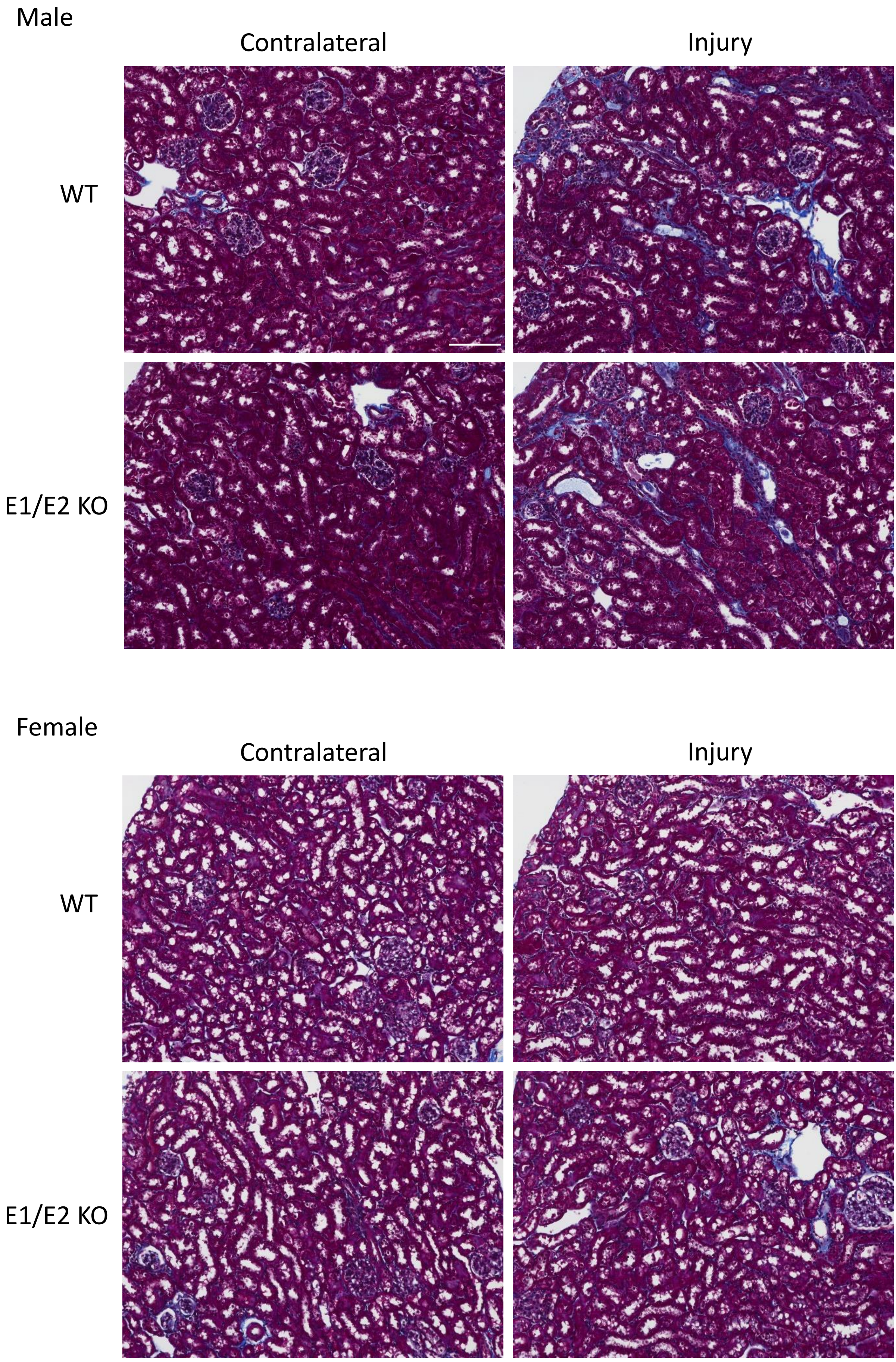

Supplemental Table 1

	**Sequence**
E1 sgRNA	CTTCTTTCAGTGTGTCGCTTAAA
E2 sgRNA 1	ACCCTGATGCACCTCTGAAG
E2 sgRNA 2	TATCAGGTGGGATGAAGCCA
E1 Forward Primer 1	GAACTCAGAAATCTGCCTGCTCC
E1 Reverse Primer 1	GAGTAGCTGGGACTATGGTAATGC
E2 Forward Primer	CTATTCAGTGGTGTCGTTGGTCC
E2 Reverse Primer	TTGTTCTGTCCACCATCTCTTCCC

Supplemental Table 2

Mouse strain	Deletion sequence
E1 KO (1744 bp del)	CTTAACTCATCTGTTGATGGACTGTTATCCTGGCTGTTGAGAACAAAGCTTCAATAAATATGGGAGTGAAACTGCCCTTTG<br>ATGTACTGAGTTATTTTCCTTTGGGACTGGGAATTGCTGGATCACATGGAAATTTGTTCTTCATATTCTTGGTGACTGCACT<br>GGTTTCCATAATGACAGGACTAATATTAAGTCATGGTTTGAAGACAGCAGTCATCTTAGAGGTGTGTGGCCCTAGCACCC<br>TTCCCTTCCTCATGTGTAGCCCTAGGAAAGCAAACAAATAGTACAGGCTTCCCTATATTAGAAAGACCACAGCTGGGCTG<br>GAGAGATGGCTCAGTGGTTAAGAGCATTAACTGCTCTTCCAAAGGTCCTAAGTTCAAATCCCAGCAACCACATGGTGGCT<br>CACAACCATCTGTAATGAGATCTGATGCCCTCTTCTGGTGTGTCTGAAGACAGCTACAGTGTATTTACATATAATAAATAA<br>ATCTTAAAAAAAAAAAAAGAAAAAAAAAAAGACCATGGTTGTGTACTCTCTCACCTCCACAAGCCATGAGGATCAAAGGA<br>GACAAACTGGGAAGACATGCTGGCAACTCAGTCACTCTGTGAATGTTTGCTGTTCTCTCAGATTGGACCTTACTCTGTATC<br>TATCAAATTTGATATCTCCAGCAAGCCTAGTGAGTGTGGTTTGTCACTTAAGAACAGACTTAATTGGGGGGGGGGCTAAA<br>TCTGGGAGTGTTAGTACTAAACATGTCTCTATGTGACTGTCTCACCAGGTCCAGGAATAATTACAAGTTTAGGGGTTGAG<br>ATTTACTTGCATAGAGAGGGTAGGGATAGGGTGGGAAGGGTAAGAGGTTAGAAGGAAGGCAAAGGGTCACATGATGT<br>CAAGTGCTGGAGAACACTGGGCAGTGTTCAGTGTGGATGGAGAGGAAGAAGTCAGGGGAATCACAGCACCCCATTCAC<br>AGCTGAGCATCAGCTGGGTCCCGTGTCTGTGCTTGAGAGAGAGTCAGTAGTCAGTAGTCCTGGTGATAGGAGATGAGCT<br>GCAGACAGAGGTGAGATACTCCCTGGCTGGCTCTCCTGCCTGAGTGCTCTCTGCACTTGCAGCCTCACGACTTTCCCTTGT<br>ACTCTTGCACATGGCCATGGTAAAGGTTAACTGTGACCACCTGTCAAAGCCTGTTATATGTAGTTCCTCTCAGCCAGGCCT<br>TATGTGAAGCCTGGGTGACTGCAGGAGGCAAAGCAGACATGGCCCCTGGACTCTTGGCACTCAGTGTCTGTGGGGGAG<br>GTACACATGAAGACACAAGGAAATGCATACCCTAGAGCCAGGACACGTTGTGGAGGAGATGCCTGGAATCCTTGAGAGT<br>TTAGAGACAGGTCTGCACACTGAAGGTGTGTGAAATAAAAATAAACAAACACACAAGAGCGGTACATTAAATTAAAAAA<br>AAAATCTTGGCAAATACAAAGTGACTAGTTCTCACAGCAAGATCAAGTCTTGCTTTTAACTGAAGATGTTGGGGTTTTATT<br>GTTTTAACAAATATGTGTGTTTTCCTCTTTCCGAGTTTGGAAAAACAATCAAGCAGACATCCCACAAAGTGCAGCCCTGGA<br>AAACCTCGCCATCTGGACTGTCTGCTGGAAAAAACAGAAAGGTCACCCAGTGTGACTTGTCGAGCTTCACTAATTCCTGG<br>AATTCCGTAGTCATTTCTCCAAATTAATTTTTAACTTCTTTCAGTGTGTCGCTTAAAATAAAATTT
E1 1001 bp del	GTGTGTCGCTTAAAATAAAATTTTTATCTCACTAAGGGGGGAAAAACAGTGGGAGGGGAAAAAGGATGGAGGGGTTGA<br>CTTAACCTTTAAGTGTTAGCAGCACACAGAATTCAAAGTGCTTTACATAAATCAGAATCAGGGAAAAGCCAGGTGAGGTA<br>GTGCATTACCATAGTCCCAGCTACTCAGGAGACTGAGCAGGAAAACCCACCCCAGTTTCAAGGCCAGCCTGCGCTACAGA<br>GTGAGTTTAATGCTAGTCCAGGCTCTGCAGAGAGTTACTTTGTAACGGACTATAGAAATGATCCCTGAAAGTATAGTCTT<br>GAGAGTTATTTTGTATATTCCTCTTATCTTTTTTTTTTTTTTTTTTTTTTTTTTTTTTTTTTGGTTTTTTTCGAGACAGGGTTTCTCT<br>GTATAGCCCTGGCTGTCCTGGAACTCACTTTGTAGACCAGGCTGGCCTCGAACTCAGATTCACCTGCCTCTGCCTCCCGAG<br>TGCTGGGATTAAAGGCGTGCACCACCACGCCCGGCCTTATTCCTCTTATCTTAGTATCTCCCAGAATTCCATGCCCCCTTAA<br>CTACAGGTAAAAGTGAGACTTATCTACAGTAAAGCTACAGTGTGAAGCTCCTGGGCTGTCTTTGTCAACTTCTTCTCCACT<br>CACATCATATTTGTTAATGAAACCTTTAGTGTTTATTCATTTATTTTATGTGTACAGATGCTTTTTGTTCTCTGGCATTTATAT<br>ATGTGCACCATATCCATACAGTGTCCATTGGGGCCAGGCAAGGGTGTCAGATCCTATGAAACTGATTAAGGACAGTTTTG<br>AGACATCCTGAGGGTGTTGGGAAGTGGACCCAGGTCCTCTGAAAGAGCAGCCAGTGCTTTTAACTGCTAAGCCATCTCTC<br>ATGCCCAGCCATGGTCTTTCTAGTACAGGGAAGCCTGTGATAGAAGCACCTTCTATAGAAAGTGTCCAGTGAGTGTAGCT<br>AGTGACTTCTAAGGTCAGTGGGTGTCTCA
E1 145 bp del	ATCTGGACTGTCTGCTGGAAAAAACAGAAAGGTCACCCAGTGTGACTTGTCGAGCTTCACTAATTCCTGGAATTCCGTAG<br>TCATTTCTCCAAATTAATTTTTAACTTCTTTCAGTGTGTCGCTTAAAATAAAATTTTTATCTCAC
E1 31 bp del	ATTTTTAACTTCTTTCAGTGTGTCGCTTAAA
E2 KO	ACCCTGATGCACCTCTGAAGAGGAGGTCAGAGAGTTCACAGGTAAAAGACGAGGTTGGAAATACTTTTAAAGAATACAA<br>GTTCACGGACCAGTGAGCCGCGGTTATGCATTTAGGAGCCGTCGCCTTTTGCGAGTGACAACTCACTGTCCACGTACTTG<br>GGCAGGAGAGATAGAAGTTAATAAATGAACACGAAGGGACCCAGAAAAGAGCATTGCTGATAGAACACAGCAGAGCTG<br>GGTGGATGCCAAACAGCCATGGGCACACCGCAGCGAAAGGAGGAACAACAGAAGCGCTTTCCAACAAATAACCAACAA<br>ATTTGGTGCGGAGGGCAGAGAGACGACTGCTGAAGGGAGCTGATCGTTGTGCTGGGCCAGGAGGATTAAGATGAGTTT<br>GCTGCTTATATCCAGACACTGTGCCATGTGCTCCCAGCTCGAGGGGGCTGAAGCCAGAACGCTGAGCAAGCCCAGCATG<br>ACATGTGCACAGACGTGGTCCCTCTCCCTCTGCAAGAGAAGCCCCAATGCCTTCCTGGTTCCAAATCGCCTGAAATTGAG<br>GTGTATGAAACTGGGTACTGGCTGGTAAATTTACCTTAGTAAGGCACGCTCTGAACTCCACTGCCGGTCAAAGGTGGATT<br>AATTCAGATGACCTGGAGGGAAAGTTCACACGAATCACCTCTCTCTCTCTCTCTCTCTCTCTCTCTCTCTCTCTCTCGCTC<br>TCGCTCTCGCTCTCTCGCTCTCTCTTGCTTTCTCTCTCTTGCTCTGTCTCTGTCTCTCTCTCTCTCTCGCTCTCTCGTTCTCTC<br>GCTCTCTCGCTCTCTCGCTCTCTCGCTCTCTCGCTCTCTCGCTCTCTCGCTCTCTCGCTCTCGCTCTCGTTCTCATCTCATCCC<br>ATGGCAGATGGGCGCACTCTGTTGTGGCTACAAAACTCAGGAACGCCCAGGCTTGCTAATTCAGGTATCAGGTGGGATG<br>AAGCCAAGGG
E1/E2 KO (E2)	CGAGGTTGGAAATACTTTTAAAGAATACAAGTTCACGGACCAGTGAGCCGCGGTTATGCATTTAGGAGCCGTCGCCTTTT<br>GCGAGTGACAACTCACTGTCCACGTACTTGGGCAGGAGAGATAGAAGTTAATAAATGAACACGAAGGGACCCAGAAAA<br>GAGCATTGCTGATAGAACACAGCAGAGCTGGGTGGATGCCAAACAGCCATGGGCACACCGCAGCGAAAGGAGGAACAA<br>CAGAAGCGCTTTCCAACAAATAACCAACAAATTTGGTGCGGAGGGCAGAGAGACGACTGCTGAAGGGAGCTGATCGTTG<br>TGCTGGGCCAGGAGGATTAAGATGAGTTTGCTGCTTATATCCAGACACTGTGCCATGTGCTCCCAGCTCGAGGGGGCTG<br>AAGCCAGAACGCTGAGCAAGCCCAGCATGACATGTGCACAGACGTGGTCCCTCTCCCTCTGCAAGAGAAGCCCCAATGC<br>CTTCCTGGTTCCAAATCGCCTGAAATTGAGGTGTATGAAACTGGGTACTGGCTGGTAAATTTACCTTAGTAAGGCACGCTC<br>TGAACTCCACTGCCGGTCAAAGGTGGATTAATTCAGATGACCTGGAGGGAAAGTTCACACGAATCACCTCTCTCTCTCTCT<br>CTCTCTCTCTCTCTCTCTCTCTCTCGCTCTCGCTCTCGCTCTCTCGCTCTCTCTTGCTTTCTCTCTCTTGCTCTGTCTCTGTCT<br>CTCTCTCTCTCTCTCGCTCTCTCGTTCTCTCGCTCTCTCGCTCTCTCGCTCTCTCGCTCTCTCGCTCTCTCGCTCTCTCGCTCTC<br>TCGCTCTCGCTCTCGTTCTCATCTCATCCCATGGCAGATGGGCGCACTCTGTTGTGGCT
